# Detection and identification by PCR of *Clostridium chauvoei *in clinical isolates, bovine faeces and substrates from biogas plant

**DOI:** 10.1186/1751-0147-51-8

**Published:** 2009-03-03

**Authors:** E Bagge, S Sternberg Lewerin, K-E Johansson

**Affiliations:** 1 Department of Bacteriology, National Veterinary Institute, SE-751 89 Uppsala, Sweden; 2 Department of Biomedical Sciences and Veterinary Public Health, Swedish University of Agricultural Sciences, SE-751 89 Uppsala, Sweden; 3 Department of Disease Control, Section for Epizootiology, National Veterinary Institute, SE-751 89 Uppsala, Sweden

## Abstract

**Background:**

*Clostridium chauvoei *causes blackleg, an acute disease associated with high mortality in ruminants. The apparent primary port of entry is oral, during grazing on pasture contaminated by spores. Cases of blackleg can occur year after year on contaminated pastures. A method to determine the prevalence of *C. chauvoei *spores on pasture would be useful.

The standard method for *C. chauvoei *detection is culture and biochemical identification, which requires a pure culture. In most muscle samples from cattle dead from blackleg the amount of *C. chauvoei *in samples is high and the bacterium can easily be cultured, although some samples may be contaminated. Detection by PCR would be faster and independent of contaminating flora.

Digested residues from biogas plants provide an excellent fertiliser, but it is known that spore-forming baeria such as *Clostridium *spp. are not reduced by pasteurisation. The use of digested residues as fertiliser may contribute to the spread of *C. chauvoei*. Soil, manure and substrate from biogas plants are contaminated with other anaerobic bacteria which outgrow *C. chauvoei*. Therefore, detection by PCR is would be useful. This study applied a PCR-based method to detect of *C. chauvoei *in 25 muscle and blood samples, 114 manure samples, 84 soil samples and 33 samples from the biogas process.

**Methods:**

Muscle tissues from suspected cases of blackleg were analysed both by the standard culture method followed by biochemical identification and by PCR, with and without preculture. To investigate whether muscle tissue samples are necessary, samples taken by swabs were also investigated. Samples from a biogas plant and manure and soil from farms were analysed by culture followed by PCR. The farms had proven cases of blackleg. For detection of *C. chauvoei *in the samples, a specific PCR primer pair complementary to the spacer region of the 16S-23S rRNA gene was used.

**Results:**

*Clostridium chauvoei *was detected in 32% of muscle samples analysed by culture with identification by biochemical methods and in 56% of cases by culture in combination with PCR. *Clostridium chauvoei *was detected in 3 (out of 11) samples from the biogas plants collected before pasteurisation, but samples taken after pasteurisation and after digestion all tested negative. *Clostridium chauvoei *was not detected in any soil or silage samples and only one manure samples tested positive.

**Conclusion:**

The diagnostic method used for *C. chauvoei *was not applicable in estimating the risk of blackleg on particular pastures from manure or soil samples, but found to be highly useful for clinical samples.

## Background

*Clostridium chauvoei *belongs to the histiotoxic clostridia and causes blackleg, a disease associated with high mortality in cattle and sheep. Animals other than ruminants are rarely infected [1-3]. Young growing ruminants on pasture are especially sensitive to *C. chauvoei*. In endemic areas *C. chauvoei *may be present in soil and faeces [2,4,5]. Once pastures have become heavily contaminated, cases of the disease usually occur year after year in susceptible animals [2]. The infection appears to be transmitted by the oral route during grazing or when eating silage or hay contaminated by spores.

Isolation of *C. chauvoei *is difficult, since it must be cultured under strict anaerobic conditions, and samples are frequently contaminated with other anaerobic bacteria that outgrow *C. chauvoei *[6]. The standard method for detection of *C. chauvoei *is based on culture and in most muscle samples from cattle dead from blackleg the amount of *C. chauvoei *is high. However, this is not always the case and contaminant flora may hamper culture. Before biochemical identification, it is important to have a pure culture of the strain. A fast and reliable detection system for *C. chauvoei *is desirable in samples containing a mixed flora. Samples from blackleg cases are sent to the laboratory as muscle tissue, since this is the easiest material from which to extract pure culture. A method not requiring pure culture, samples could be sent to the laboratory as swabs from infected muscle tissues.

Manure can contain bacterial spores and possibly also vegetative bacteria that pass through the digestive systems of animals grazing on contaminated pasture. Manure is part of the substrate for biogas plants, and spore-forming bacteria may survive pasteurisation and digestion [7,8]. An anaerobic digestion process is commonly used for energy production in the form of biogas. The digested residues produced are an excellent fertiliser, rich in plant nutrients, and application of digested residues to agricultural land reduces the need for artificial fertilisers. However, organic wastes contain many different types of biological contaminants. To reduce the risk of spreading pathogens, the recommended biowaste treatment method before anaerobic digestion is heating to 70°C for 60 min if manure and animal by-products are present in the substrate, as regulated by EU Commission regulation EC no 1774/2002 and 208/2006. Heating at 70°C for 60 min reduces *Salmonella *spp. [8,9]. However, some spore-forming bacteria such as *Clostridium *spp. and *Bacillus *spp. are not eradicated [7-10].

Manure and animal by-products from slaughterhouses are sent to local biogas plants. If cattle from high-risk areas shed *C. chauvoei *in faeces or if animal by-products are contaminated with *C. chauvoei*, it is possible to spread the bacteria to previously unaffected areas via biogas plants if contaminated digested residues are spread in areas free from *C. chauvoei*. In soil, spores can survive for many years [11].

In certain areas of south-east Sweden, blackleg is regarded as an endemic disease [12]. A method to determine the prevalence of *C. chauvoei *spores on pasture could also serve as an indication of the risk of blackleg, enabling farmers to design herd-specific vaccination programmes. In areas where blackleg is endemic, it is both an economic problem for farmers and an animal health problem. Affected farms usually have vaccination routines, but farmers seek to minimise the use of vaccine [3,12]. It would help farmers to know where on their farm the prevalence of *C. chauvoei *is high, in order to focus vaccination routines on animals grazing high-risk pastures.

The aim of this study was to investigate the suitability of using PCR for detecting *C. chauvoei *in muscle tissue taken at autopsy and investigate the prevalence of *C. chauvoei *in faeces, soil, biogas substrate and digested residues samples.

## Methods

### Collection of samples from suspected cases of blackleg

One blood sample from 2005, five muscle samples from 2006, six muscle samples from 2007 and 13 muscle samples from 2008 were included in the study. Muscle tissues and a blood sample from cattle with clinical symptoms of suspected blackleg were sent by ordinary postal services to the bacteriological laboratory at the National Veterinary Institute (SVA). Muscle tissue samples were transported in sealed plastic bags and blood sample in a Vacutainer^® ^tube. These samples were cultured anaerobically at 37°C for 48 h on Fastidious Anaerobic Agar plates (FAA, LabM, Bury, Lancashire, England), with 5% defibrinated horse blood. The bacteria isolated were identified by biochemical methods according to standard methods at SVA, which comprise fermentation of glucose, maltose, lactose, sucrose, starch, mannitol and fructose, and production of lecithinase, tryptophanase, urease and hydrolysis of aesculin. One loopful (approximately 5 μL of bacteria collected with a 10 μL loop) was collected from each FAA plate by a streak over the swarming flora (mixed, non-discrete colonies). The loopful was used for DNA preparation followed by PCR detection. After routine analysis for *C. chauvoei*, the muscle samples were stored at -20°C and the blood sample was taken for DNA preparation directly.

The following samples was used for DNA preparation followed by PCR detection

a) Muscle piece, approximately 1 g.

b) A muscle piece, approximately 1 g, added to 500 μL of sterile physiological saline, macerated and left in the water for 5 min. The muscle piece was then removed from the water, and the remaining water was analysed.

c) Meat juice (500 μL), collected from the bottom of the muscle tissue storage bottle.

d) Blood sample (500 μL).

#### Muscle samples collected by swabs

Specimens were taken from most of the muscle tissues (22) by swabs (Amies' medium with charcoal, Nordic Biolabs, Täby Sweden) and a postal service comprising 1, 3 and 6 days (3 swabs/-muscle tissue) was simulated. The samples were taken in the centre of the muscle tissue and after sampling the swabs were left on a bench at room temperature (approximately 20°C) during the simulated postal service. After 1, 3 and 6 days the swabs were streaked onto FAA plates and incubated at 37°C for 48 h under anaerobic conditions. One loopful of colony material was used for DNA preparation.

### Collection of samples from biogas plants

Samples from a biogas plant located in south-east Sweden were taken weekly at 11 different occasions before pasteurisation, after pasteurisation and after digestion. None of farms A-K tested (see below) sent manure to the biogas plant, but these farms did send their animals to the local slaughterhouse. This slaughterhouse sent animal by-products to the biogas plant. Approximately 200 g of samples were collected in clean pots on each sampling occasion. The samples were stored at -20°C until analysis.

Five g of each biogas plant sample were placed in a separate bottle and 10 mL of sterile physiological saline were added. The bottles were shaken thoroughly and left for 30 min., allowing the material to settle. Two mL of the supernatant were heated at 65°C for 10 min and two mL were enriched in Fastidious Anaerobic Broth (FAB, LabM, Bury, Lancashire, England), and heated at 65°C for 10 min. An aliquot from each one was then streaked onto FAA plates and incubated in an anaerobic jar at 37°C for 48 h. One loopful of colony material was collected for DNA preparation.

### Collection of samples from farms

#### Manure, soil and silage

Eleven dairy farms (A-K) from the island of Öland of south-east Sweden were included in this study (Table 1). Blackleg is endemic on this island [12]. All samples from farms A-I were collected at one occasion during a visit to the island. Farms J and K reported cases of blackleg after the visit and were subsequently included in the study. *Clostridium chauvoei *had been detected in muscle samples from these two farms collected at necropsy. On farms G, H, I and J, individual faecal samples were taken from the floor behind housed cows. On all other farms, the faeces samples were collected outdoors as cowpats on grassland. Soil samples were collected from all farms. Depending on the ground, grassland or soil, between three to eleven samples were collected on each farm. Two farms (A and B) grazed their animals on a common pasture by the seashore. For these farms, only three soil samples were taken around the cattle house and eleven soil samples were taken from the common pasture. All samples were collected during the grazing period. In total, 114 faecal samples, 84 soil samples and four silage samples were collected for analysis. The weight of each the sample was approximately 100–200 g and they were collected in clean pots. The samples were stored at -20°C until further analysis.

**Table 1:** Description of farms A-K from which samples were obtained for the study

**Farm**	**A**	**B**	**C**	**D**	**E**	**F**	**G**	**H**	**I**	**J**	**K**
**No. cows**	130	72	50	24	120	40	70	50	130	120	nk
**No. heifers**	150	140	80	30	130	80	100	45	260	nk	nk
**Cases of blackleg**	14	nk	1	2	5–6	5–6	10	10	6–8	1–2	3
**Year of occurrence**	2002	2002	2001	2001	2003	2001	2000 2001 2002	2004	2003 2004	2005 2006	2006
**Affected animals**	heifers	calves, heifers	heifers	heifers	heifers	1 cow 4–5 heifers	heifers	calves	heifers	heifers	nk
**Vaccination performed**	yes	yes	yes	yes	yes	yes	yes	no	no	no	no
**No. of faeces samples**	10	10	10	10	10	7	10	10	10	20	7
**No. of soil samples**	3*	3*	3	9	5	8	10	6	11	11	4
**No. of silage samples**	-	-	-	-	-	-	-	-	-	-	4

nk = Not known

* For farms A and B, 11 soil samples were taken from a common pasture by the seashore plus three soil samples were taken around each cattle house.

Five g of each sample of manure, soil and silage were placed in a separate bottle, 10 mL of sterile physiological saline were added and these samples were prepared by the same procedure as the biogas samples (See previous chapter).

#### Data collection from farms

On each sampling occasion, data concerning herd size, vaccination routines, number of samples and history of blackleg were collected using a questionnaire (Table 1). Data were also collected on other aspects such as feeding, manure-spreading practices and grazing routines (data not shown).

### DNA preparation

During preparations for this project, four different DNA preparation methods were evaluated. The DNA preparation methods were phenol/chloroform, boiled lysate and two commercial kits (UtraClean™ Soil DNA Isolation Kit, Mo Bio labs, Solana Beach, California, USA and Genomic DNA purification Kit, Fermentas, Burlington, Canada) for which the manufactures' recommendations were followed.

The colony material from FAA plates, blood, muscle pieces, muscle pieces macerated in sterile physiological saline and meat juice were suspended in 500 μL of phosphate-buffered saline (PBS, Merck, Darmstadt, Germany) and centrifuged for 10 min at 7,200 × g. The supernatant was discarded and the pellet was washed again by the same procedure. The pellet was thereafter re-suspended in 250 μL of sterile H_2_O. The bacterial cells were lysed by boiling the suspension for 15 min before storage at -20°C until further analysis. This lysate was used as template in PCR.

### PCR

For detection and identification of *C. chauvoei *in the samples, a specific PCR primer pair complementary to the spacer region of the 16S-23S rRNA gene was used as described by Sasaki *et al*. [13,14].

The amplicons were analysed by electrophoresis in agarose gels (1.5% Agarose NA, from GE Healthcare, Uppsala, Sweden) which were stained with ethidium bromide. The PCR products were visualised under UV-light. The size of the amplicon was 509 bp [13]. Boiled lysate of *C. chauvoei *AN 2548/02 was used as a positive control. Closely related clostridia such as *Clostridium septicum *and *Clostridium perfringens*, which can occur as contaminants in clinical infections of *C. chauvoei*, were used as negative controls.

To confirm that the PCR product of *C. chauvoei *AN 2548/02 originated from *C. chauvoei*, further analysis was performed by 16S rRNA gene sequencing. The PCR product was diluted and cycle sequencing reactions were carried out with BigDye Terminator v3.1 Cycle Sequencing Kit (Applied Biosystems, Foster City, CA, USA) according to the manufacturer's recommendations. The nucleotide sequences were determined with the ABI PRISM 3100 Genetic Analyzer (Applied Biosystems). The sequences were merged into one contig using the programme ContigExpress, Vector NTI suite ver. 9.0. To compare the sequences, similarity searches were done in GenBank [15].

### Detection threshold of the PCR method

#### Preparation of the strain

One loopful of *C. chauvoei *(AN 2548/02) was inoculated into 10 mL of serum broth. The broth was incubated at 37°C for 48 h under anaerobic conditions. In order to stimulate the production of spores, the broth was then kept at room temperature for further 5 days as described by Båverud *et al*. [16]. The concentration of bacteria was checked by the viable count method. The numbers of bacteria were quantitatively analysed using a ten-fold dilution series in peptone saline solution, which was then streaked (0.1 mL) onto FAA plates and incubated anaerobically at 37°C for 48 h. After incubation the number of colonies was counted. The quantitative dilution experiment was performed twice.

#### *Clostridium chauvoei *spiked samples

In order to determine the detection level of the PCR method spiked samples of cattle manure, soil, silage and substrate from a biogas plant were analysed. The samples from the biogas plant were from both before and after pasteurisation and from digested residues. Soil and manure were taken from an area free of blackleg as well as the biogas plant samples.

From the bacterial suspensions, 10-fold dilutions were made in peptone saline solution. One mL from each dilution step between 1 and 7 was thoroughly mixed with 5 g of from faeces, soil or silage substrate. Five g of biogas substrate before and after pasteurisation were mixed with 1 mL of the bacterial suspension from each dilution step between 1–5 and digested residue was mixed with bacterial suspension from dilution steps between 1 and 6. All bottles were agitated and then left to settle for 30 min. The detection thresholds for *C. chauvoei *for each stage in the biogas process were estimated using ten-fold dilutions in peptone saline solution followed by total viable counts onto FAA plates (0.1 mL) incubated at 37°C for 48 h under anaerobic conditions. For confirmation of the strains, one loopful from each agar plate was used for DNA preparation after incubation. All dilution series were made in duplicate.

## Results

### Samples from suspected cases of blackleg

*Clostridium chauvoei *was detected in 8 out of 25 (32%) of the samples from the suspected cases of blackleg analysed by culture methods and identification by biochemical methods. For PCR in combination with culture on FAA plates, 14 out of 25 (56%) of samples were above the detection threshold. In muscle pieces or blood without any preculture 3 out of 24 (12%) samples were above the detection threshold of PCR, while in meat juice 2 out of 23 (9%) samples were above the detection threshold and in muscle pieces macerated in sterile physiological saline 6 out of 23 (26%) samples were above the detection threshold (Table 2).

**Table 2:** Comparisons of *Clostridium chauvoei *detected by different methods

Sample	Material	Culture followed by biochemical identification	PCR on culture colonies	PCR on muscle piece or blood	PCR on meat juice	PCR on muscle piece macerated in sterile physiological saline
AN 3656/05	blood	+	+	-	nd	nd
AN 165/06	muscle	+	+	+	-	+
AN 1106/06	muscle	+	+	-	-	+
AN 1717/06	muscle	+	+	-	-	-
AN 1818/06	muscle	-	-	-	-	-
AN 2091/06	muscle	-	+	-	-	-
B 16476/07	muscle	-	+	+	-	-
B 17520/07	muscle	-	-	-	-	-
B 19034/07	muscle	-	-	-	-	-
P 2500/07	muscle	+	+	nd	nd	nd
P 2855/07	muscle	-	+	-	+	+
P 4822/07	muscle	-	-	-	-	-
P 2446/08	muscle	-	+	-	-	-
B 29245/08	muscle	-	+	-	-	+
B 32286/08	muscle	-	-	-	-	-
P 3086/08	muscle	+	+	-	-	-
P 3240/08	muscle	-	-	-	-	-
B 41901/08	muscle	-	-	-	-	-
B 41902/08	muscle	-	-	-	-	-
B 43014/08	muscle	-	-	-	-	-
B 43531/08	muscle	-	-	-	-	+
P 3837/08	muscle	-	-	+	-	+
B 46320/08	muscle	+	+	-	-	-
P 4324/08	muscle	-	+	-	+	-
P 4325/08	muscle	+	+	-	-	-

Summary		8+/25	14+/25	3+/24	2+/23	6+/23

nd = Not determined

Comparisons of *Clostridium chauvoei *detected by culture followed by biochemical identification culture followed by PCR and direct PCR on muscle tissue from clinical samples of blackleg.

#### Muscle samples collected by swabs

In 22 muscle tissues cultured on FAA plates, 14 (64%) were above the detection threshold of PCR, while 11 (50%) swabs from the same 22 muscle tissues were above the detection threshold (Table 3).

**Table 3:** Comparison between muscle tissue samples and swab samples

Sample	Culture followed by biochemical identification	Summary of PCR results from Table 2	1 day	3 days	6 days
AN 165/06	+	+	-	-	-
AN 1106/06	+	+	+	+	+
AN 1717/06	+	+	-	+	-
AN 2091/06	-	+	-	-	-
B 16476/07	-	+	+	+	+
B 17520/07	-	-	-	-	-
B 19034/07	-	-	-	-	-
P 2855/07	-	+	+	+	+
P 4822/07	-	-	-	-	-
P 2446/08	-	+	-	-	-
B 29245/08	-	+	+	+	+
B 32286/08	-	-	-	-	-
P 3086/08	+	+	+	+	+
P 3240/08	-	-	-	-	-
B 41901/08	-	-	-	-	nd
B 41902/08	-	-	-	-	nd
B 43014/08	-	-	-	-	nd
B 43531/08	-	+	-	+	-
P 3837/08	-	+	-	-	+
B 46320/08	+	+	+	+	+
P 4324/08	-	+	+	-	+
P 4325/08	+	+	+	-	+

Summary	6+/22	14+/22	8+/22	8+/22	9+/19

nd = Not determined

Muscle tissue samples from suspected cases of blackleg. *Clostridium chauvoei *were analysed by culture and biochemical identification, culture and PCR and swab samples from the same muscle samples were analysed by culture and PCR. The swabs were used to simulate a postal service simulation by keeping them on the bench for 1, 3 and 6 days.

### Samples from biogas plants

Three samples from the biogas plants taken before pasteurisation were above the detection threshold of PCR for *C. chauvoei*, but samples taken after pasteurisation and after digestion were all below the detection threshold (Table 4).

**Table 4:** Detection of *Clostridium chauvoei *by PCR in manure, soil, silage and biogas plant substrate samples

**Material**	**No. of samples**	**No. of positives**
Manure	114	1
Soil	84	0
Silage	4	0
Before pasteurisation	11	3
After pasteurisation	11	0
After digestion	11	0

### Samples from farms

All field samples of faeces (114) except one, soil (84) and silage (4) were below the detection threshold of the PCR method for *C. chauvoei*. One faecal sample was above the detection threshold (Table 4).

### PCR

From the evaluated DNA preparation methods the most reliable method was boiled lysate with previous culture on FAA plates. For phenol/chloroform and the two commercial kits, the detection threshold was approximately ten times higher than for boiled lysate (data not shown). DNA concentrations were not measured in the DNA template of boiled lysate because of the mixed flora.

The positive control, *C. chauvoei*, strain AN 2548/02, was included in all PCR analyses and it always proved positive as evaluated from electrophoresis in agarose gels. PCR products from closely related clostridia such as *C. septicum *and *C. perfringens *were not detected by the specific primer pair used for detecting of *C. chauvoei*.

Sequence analysis of the 16S rRNA gene of strain AN 2548/02 and similarity searches in GenBank showed that it was identical to *C. chauvoei*, strain ATCC 10092^T ^[GenBank: U51843].

### Detection threshold of the PCR method

The detection thresholds for samples consisting of manure, soil, silage and biogas substrate before and after pasteurisation and digested residues were 200 colony forming units (cfu)/g.

## Discussion

### Samples from suspected cases of blackleg

For muscle samples from suspected cases of blackleg, the results from culture on FAA plates followed by PCR from colony material were in agreement with, or performed even better, than the biochemical identification method (Table 2). These results are consistent with Uzal *et al*. [17]. This study demonstrated that DNA preparation from culture before PCR gave better results than PCR applied directly on biomass.

Culture followed by biochemical identification methods is complicated in samples with a high content of contaminating flora because of the need for pure culture and the sensitivity was substantially lower than for culture and PCR. In eight cases it was impossible to detect *C. chauvoei *by culture and biochemical identification but the organism was detected by PCR (Table 2). For making a diagnosis of blackleg, muscle tissue is taken at autopsy since the amount of *C. chauvoei *probably is higher in muscle tissue than in blood. The single blood sample was incorrectly taken, but the question was blackleg.

Likewise, in a previous study of a blackleg outbreak, culture and detection by PCR gave better results than biochemical analysis [18]. In one of the cases, primary culture of one muscle on a blood agar plate was overgrown by *C. septicum *but gave clear positive results for *C. chauvoei *in the PCR.

In most clinical cases of blackleg the amount of *C. chauvoei *in infected tissue is high and can easily be detected by the PCR method independent of contaminating flora. Moreover, the PCR method is much faster than the traditional biochemical detection method, which takes at least five days, and it is important to have a pure culture of the strain, which might require extra time for culture. To avoid contaminating flora, the muscle tissue should not be too small; approximately 100–200 g would be adequate. Without pure culture no strains can be for subsequent studies in the future, which is a disadvantage of using PCR detection as the sole method.

In cattle experimentally infected with *C. chauvoei*, PCR analyses were carried out on minced muscle and other organs and *C. chauvoei *was detected in all organs tested [19]. Minced muscle pieces from suspected cases of blackleg were directly tested by PCR and compared with culture. The detection by PCR gave better results than culture and biochemical identification [19], which is in disagreement with the results in the present study where only 12% (3 out of 24) of the blood or muscle pieces were above the detection threshold of PCR, while 9% of the meat juice samples were above the detection threshold (Table 2). Inhibitory substances are probably present in muscle tissues. Inhibitors for the enzymatic reaction of PCR amplification have been identified in liver, spleen and kidney [20] and there may be similar inhibitors present in muscle tissue. The results from muscle pieces macerated in sterile physiological saline samples (26%) did not correspond well to those of the biochemical test. Direct PCR on muscle tissue or other similar samples is probably not suitable for replacing culture steps followed by biochemical tests or by PCR.

#### Muscle samples collected by swabs

To investigate whether it is necessary to send muscle tissue for analysis of *C. chauvoei *or whether it is sufficient to use swabs for sample collection, a postal service was simulated. Of the 22 muscle tissues investigated, 64% were above the detection threshold of PCR. For swabs from the same samples after the postal service simulation, 36% to 47% swabs were above the detection threshold (Table 3). It would be more practical to take samples with swabs instead of muscle tissues if swabs are sufficient for analysis of *C. chauvoei*. However, muscle tissues gave more positive samples than swabs when both were analysed by culture and PCR (Table 3). The incidence of *C. chauvoei *on swabs from the positive muscle tissue cases seemed to be random. However, it appeared to make little or no difference whether the swabs were stored for 1, 3 or 6 days.

### Samples from biogas plants

*Clostridium chauvoei *was detected in 3 samples out of 11 in biogas substrate before pasteurisation but it could not be detected after pasteurisation or after digestion (Table 4). The bacteria in these kinds of samples could originate from manure or from animal by-products from the slaughterhouse, as both these sources are used for biogas production. None of the farms sampled in this study send their manure to the biogas plants due to the geographical distance, but animals are sent to the local slaughterhouse. Cases of blackleg have also been reported in the catchment area of the biogas plant.

After biogas processing, the digested residues are used as fertiliser. Spore-forming bacteria can survive pasteurisation and digestion in biogas plants [7,8] and digested residues from biogas plants may therefore be capable of spreading blackleg to new areas. However, no *C. chauvoei *was detected in processed product so perhaps the environment in the digester is not suitable for *C. chauvoei*, or the flora in the digester outgrows *C. chauvoei*. Clostridial spores can survive for long periods in soil [11], which has to be taken into account in further studies on *C. chauvoei *in digester.

### Samples from farms

In spite of the fact that all farms investigated had reported suspected cases of blackleg in recent years, all samples from faeces, soil and silage tested negative for *C. chauvoei*. There could be several explanations for this. Spores could still be present in the material, but below the detection level. Moreover, PCR inhibitors may be present in the samples and the PCR can be hampered [21]. Cattle faeces contain volatile fatty acids (VFA) and the soil samples may contain traces of metal, both of which are known to interfere with the PCR reaction. However, the culture step before PCR reduces the influence of inhibitory substances.

Since *C. chauvoei *is a soil bacterium some seasonal variation may occur, for example heavy rainfall may contribute to spreading of the spores [22] and the amount of spores in the soil can be more accessible to cattle. Most sampling was carried out in early autumn when the weather was sunny and thus the concentration of spores in the samples would be expected to be low. The number of samples may have been insufficient for detection of environmental contamination. However, for practical reasons, very high numbers of environmental samples are rarely available from the field.

Suspected cases of blackleg on the farms had primarily occurred in calves and heifers but one farm reported a case in an adult cow. Seven of the eleven farms A-K had vaccination routines in place at the time of sampling (Table 1).

Two of the farms stored manure before spreading and the others did not, but none of the farms spread manure on pasture. Local veterinarians have reportedly seen more cases of blackleg after shrubbery clearing on the island of Öland, especially on Alvaret (a barren limestone plain, almost 40 km long and 10 km wide, with special flora depending on the thin soil and high pH). Alvaret is usually used as pasture by neighbouring farms. Not much shrubbery clearing had been done in the year of sampling.

### PCR

Cultural procedures are expensive and time-consuming, and contamination with other anaerobic bacteria that outgrow *C. chauvoei *frequently causes problems [17,19]. When using PCR, the DNA of both viable and non-viable cells are amplified. If only viable cells are to be detected an enrichment step can be applied [17,23]. In this study culture on FAA plates before PCR was used to avoid detection of non-viable bacteria and false positive PCR reactions. DNA is easy to amplify from pure cultures, but problems arise with contaminated samples, such as samples from biowaste. Detection of DNA by PCR can be hampered by numerous substances including humic acids, VFA, fats and proteins [21]. However, the culture step before PCR was used as an enrichment step and, therefore, reduces the influence of inhibitory substances. Due to the swarming flora, no purification from the FAA plates could be done.

### Detection threshold of the PCR method

The detection level was 200 cfu/g. This may seem to be a poor detection level but it can be explained by the fact that samples such as manure, soil and biogas substrate are heavily contaminated by the surrounding flora. Sasaki [19] reported a detection level of 10 cfu/g, apparently in cleaner samples. However, since the detection level in our study was 200 cfu/g and since such samples were expected to contain a low numbers of *C. chauvoei*. Thus, the method used in the present study is not practically applicable.

### Environmental aspects

The detection threshold of the PCR- method after preculture is hardly at current stage suitable for guaranteeing that animals are free from infection or for determining the status of pastures. Instead the currently applied recommendations have to be used, since the method cannot be used as a basis for vaccination routines. In areas where blackleg is endemic, annual vaccination before grazing is recommended, in spite of the costs and the time required.

This study gave some indication that *C. chauvoei *does not pass through the biogas process. The number of *C. chauvoei *in biowaste perhaps decreased in the digester to below the detection threshold of the method and the risk of spreading digested residues is thus negligible. More studies about clostridial survival through the biogas process are needed before definite conclusions can be drawn. However, the advantages of digested residues as a fertiliser perhaps outweigh the risk of spreading blackleg.

## Conclusion

In samples of affected muscular tissue taken at autopsy culture in combination by PCR seemed to be faster, simpler and safer than the conventional analysis by culture and biochemical identification of *C. chauvoei*. However, the corresponding use of PCR on manure and soil samples is not practically applicable as a possible tool for detecting *C. chauvoei *before moving or selling animals from contaminated areas to disease-free areas or for determining the risk of blackleg on particular pastures.

## Competing interests

The authors declare that they have no competing interests.

## Authors' contributions

EB and SSL designed the study and EB performed the practical analyses and writing the manuscript, with some assistance from SSL. KEJ was responsible for the PCR and sequence methods, and discussed and approved the final manuscript.
